# Supplementary material for: Differential interactions of ToLCNDV with different betasatellites reveal complex viral dynamics in N. benthamiana
Source: PLoS One. 2025 Jun 25;20(6):e0327234. doi: 10.1371/journal.pone.0327234 (PMC12193804; doi:10.1371/journal.pone.0327234)
Supplement: S1 File — S1 Table. List of betasatellites found associated with ToLCNDV in Solanaceous crops. S2 Table. Oligonucleotide primers used in the study for quantification and Southern blotting. S3 Table. Rep-DNA docked complexes and their binding free energy. S1 Fig. Diagram showing the positions of primers on ToLCNDV DNA A (A), DNA B (B) and TbLCB (C) that were used in the quantitative PCR assays. S2 Fig. Standard curve and melt curve for amplification of ToLCNDV DNA A. (A) Standard curve for estimation of the titre of ToLCNDV DNA A with a PCR efficiency of 96.5%, slope of −3.376 and correlation coefficient 0.992 [left] and a melt curve analysis of the products of amplification [right]. (B) Standard curve for estimation of the titre of ToLCNDV DNA B with a PCR efficiency of 95.0%, slope of −3.376 and correlation coefficient 0.991 [left] and a melt curve analysis of the products of amplification [right]. (C) Standard curve for estimation of the titre of betasatellites with a PCR efficiency of 97.0%, slope of −3.376 and correlation coefficient 0.995 [left] and a melt curve analysis of the products of amplification [right]. The standard curves plot log of starting DNA quantity against threshold cycle [C(T)] cycle. The melt curves plots negative rate of change of fluorescence [-d(RFU)/dT] against temperature. S3 Fig. Alignment of coding region βC1 (A) and the satellite conserved region (SCR) (B) from three betasatellites: tobacco leaf curl betasatellite (Tbβ), cotton leaf curl Multan betasatellite (Mβ), and cotton leaf curl Multan betasatellite strain Burewala (Bβ). Sequence similarity is indicated by color: red for highly similar regions across all three betasatellites, green for regions similar in two betasatellites, and blue for dissimilar sequences. Identified repetitive motifs are enclosed in boxes: green for motifs common to all three betasatellites, brown for motifs shared by Tbβ and Bβ, and blue for motifs shared by Bβ and Mβ. Yellow boxes denote previously reported repetitiv [file pone.0327234.s001.docx]

Supplementary Material

# Differential interactions of ToLCNDV with different betasatellites reveal complex viral dynamics in *N. benthamiana* plants

# Zafar Iqbal^1†*^, Muhammad Shafiq^2†*^, Sajed Ali^2^, Mudassar Fareed Awan^2^, Muhammad Farhan Sarwar^2^, Imran Amin^3^, Muhammad Shafiq Shahid^4^, Rob W. Briddon^3^

# ^1^ Central Laboratories, King Faisal University, P.O. Box 55110, Al-Ahsa, Saudi Arabia; zafar@kfu.edu.sa | zafariqbal2009@gmail.com (Z.I)

# ^2^ Department of Biotechnology, University of Management and Technology, Sialkot Campus, Sialkot P.O. Box 51340, Pakistan; shafiq.4721@gmail.com (M.S); sajed.ali@skt.umt.edu.pk (S.A); mudas-sar.fareed@skt.umt.edu.pk (M.F); farhan.sarwar@skt.umt.edu.pk (F.S)

# ^3^ Agricultural Biotechnology Division, National Institute for Biotechnology and Genetic Engineering Faisala-bad, P.O. Box 38000, Pakistan, imranamin1@yahoo.com (I.A.); rob.briddon@gmail.com (R.W.B.)

# ^4^ Department of Plant Sciences, College of Agricultural and Marine Sciences, Sultan Qaboos University, Al-Khoud, Muscat, Oman., mshahid@squ.edu.om (M.S.S)

# ^†^ Equal contributions

# ^*^ Correspondence: zafar@kfu.edu.sa | zafariqbal2009@gmail.com; Tel.: +966580776536; ORCID ID. https://orcid.org/0000-0001-7185-4060

#

# S1 Table: List of betasatellites found associated with ToLCNDV in Solanaceous crops.

| **Betasatellite Name** | **Host** | **Accession No.** |
| --- | --- | --- |
| Ageratum yellow vein betasatellite | Tomato | AJ542495 |
| Chilli leaf curl betasatellite | Chilli | AJ316032 |
| Cotton leaf curl Multan betasatellite | Tomato | AJ316035 |
| Cotton leaf curl Multan betasatellite | Tomato | AY438562 |
| Tobacco leaf curl betasatellite | Tobacco | AJ316033 |
| Tomato leaf curl betasatellite | Tomato | AJ542490 |
| Tomato leaf curl betasatellite | Tomato | AJ316036 |
| Tomato leaf curl Bangalore betasatellite | Tomato | AY428768 |
| Tomato leaf curl Bangalore betasatellite | Tomato | AY438560 |
| Tomato leaf curl Bangladesh betasatellite | Tomato | AJ542489 |
| Tomato leaf curl Bangladesh betasatellite | Tomato | AY438558 |

# S2 Table: Oligonucleotide primers used in the study for quantification and Southern blotting.

| Primer name | Sequence (5’-3’) | Amplicon (bp) |
| --- | --- | --- |
| For quantification through real time PCR | | |
| V2-F | GTCGAAGCGACCAGCAGATAT | 190 |
| V2-R | GGAACATCTGGACTTCTGTAC |  |
| βC1-F | GTTGTATGCGAATAGGAAATTCG | 192 |
| βC1-R | CAGGTTCATAGTCGACGTTCGC |  |
| B MP-F | GCCCATGATTCGTTCGGAC | 181 |
| B MP-R1 | GAATTCCGACCACCAAAGAT |  |
| For Southern blot | | |
| ND-B-F | GCCCATGATTCGTTCGGAC | 475 |
| ND-B-R | CACGTGGTACTGGAATATCGCA |  |
| ND-A-F | CCTTTAATCATGACTGGCTT | 249 |
| ND-A-R | CATTTCCATCCGAACATTC |  |
| Beta-F | GATTTGACTTATATTGGGCCAATTTAAT | 406 |
| Beta-R | GATACTATCCACAAAGTCACCATCGCTAAT |  |
| TbB-F | GTCTATTACAGGAGCCTCTTCCATC | 475 |
| TbB-R | CCAGAAGGGGATGGAATTCATCAT |  |
| 18s-F | TCTGCCCTATCAACTTTCGATGGTA | 137 |
| 18s-R | AATTTGCGCGCCTGCTGCCTTCCTT |  |

# Abbreviations used in the table are ToLCNDV (ND), tobacco leaf curl betasatellite (TbB), and movement protein-encoded on TB of ToLCNDV (B MP).

**S3 Table.** Rep-DNA docked complexes and their binding energy.

| **Names of Docked complexes** | **Binding Energy (kJ.mol^‒1^)** |
| --- | --- |
| Rep-TA | −241.17 |
| Rep-Tbβ | −189.72 |
| Rep-TB | −184.60 |
| Rep-Bβ | −184.11 |
| Rep-Mβ | −184.09 |

#
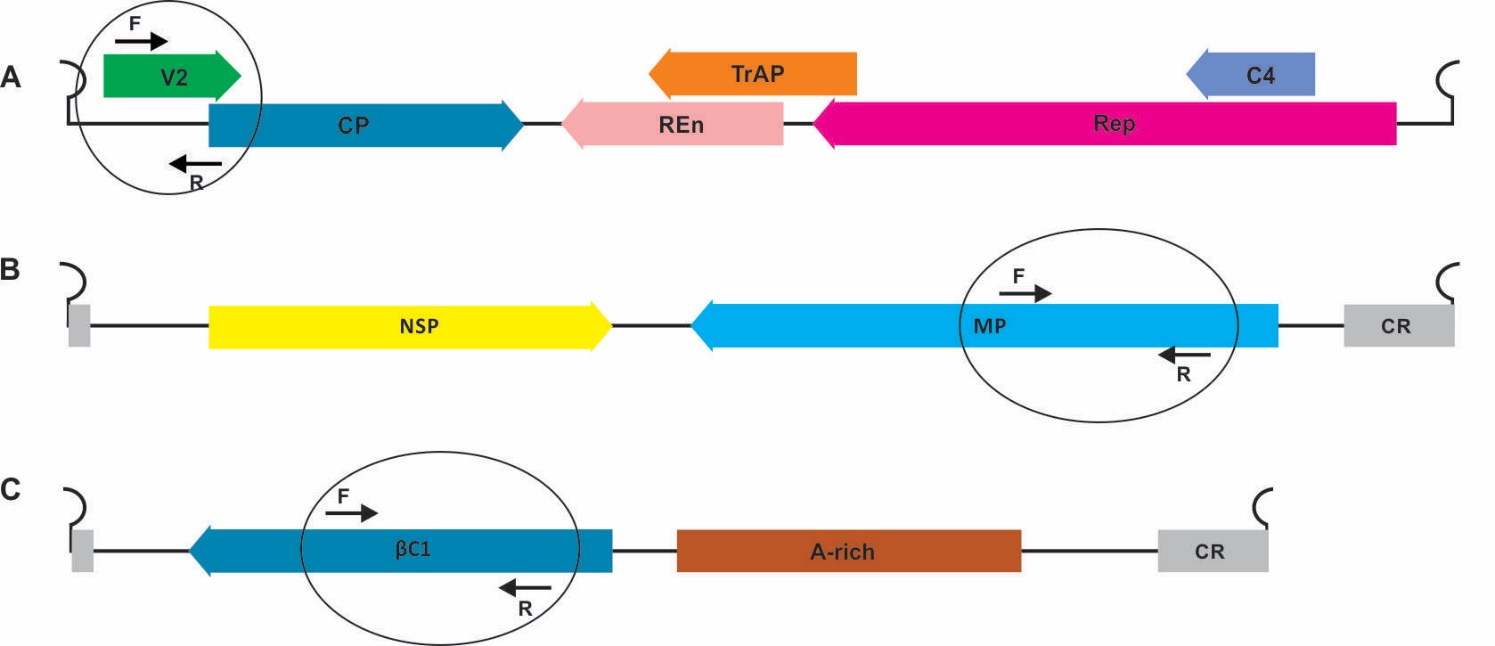


# S1 Fig. Diagram showing the positions of primers on ToLCNDV DNA A (A), DNA B (B) and TbLCB (C) that were used in the quantitative PCR assays.

#
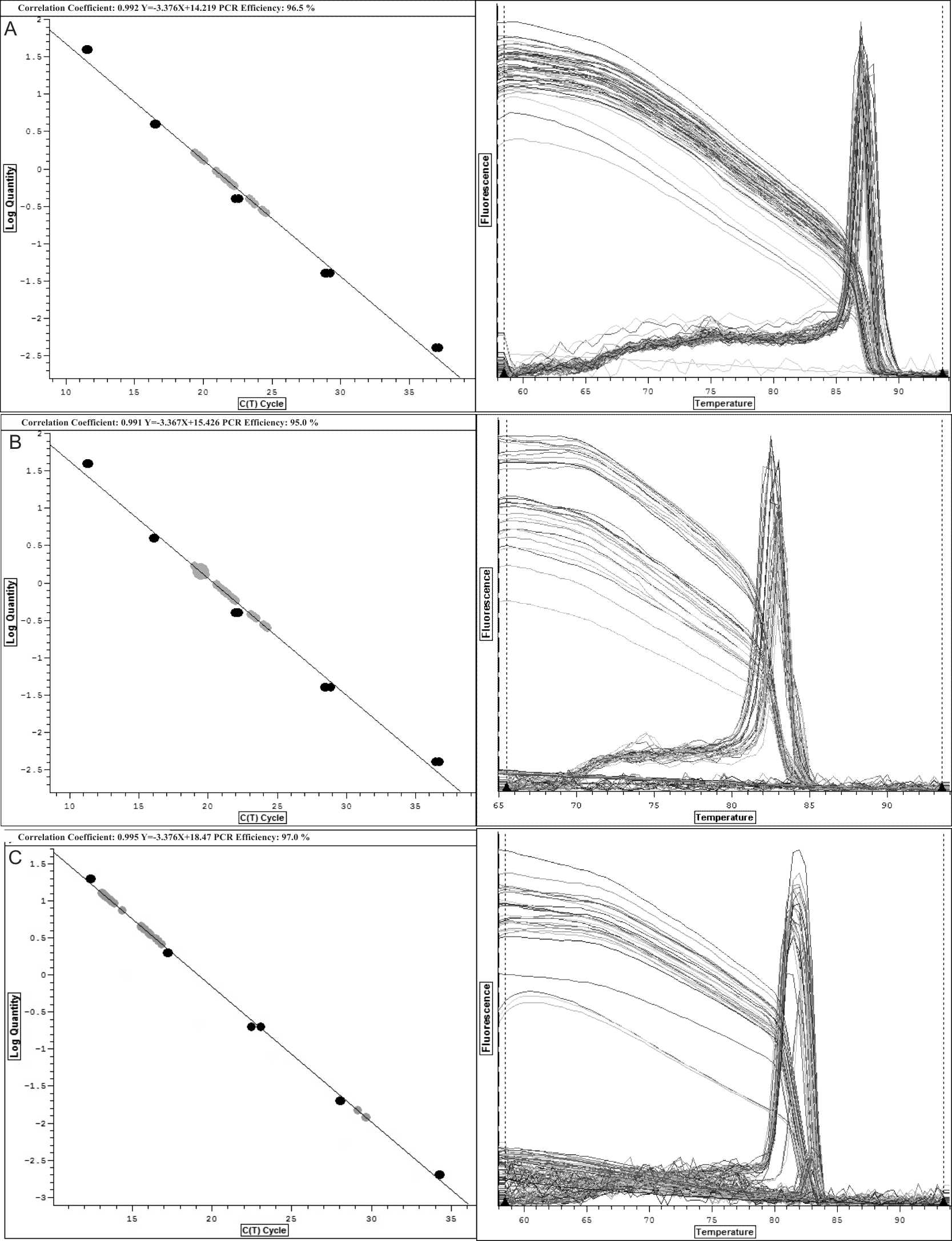


# S2 Fig. Standard curve and melt curve for amplification of ToLCNDV DNA A. (A) Standard curve for estimation of the titre of ToLCNDV DNA A with a PCR efficiency of 96.5 %, slope of -3.376 and correlation coefficient 0.992 [left] and a melt curve analysis of the products of amplification [right]. (B) Standard curve for estimation of the titre of ToLCNDV DNA B with a PCR efficiency of 95.0 %, slope of -3.376 and correlation coefficient 0.991 [left] and a melt curve analysis of the products of amplification [right]. (C) Standard curve for estimation of the titre of betasatellites with a PCR efficiency of 97.0 %, slope of -3.376 and correlation coefficient 0.995 [left] and a melt curve analysis of the products of amplification [right]. The standard curves plot log of starting DNA quantity against threshold cycle [C(T)] cycle. The melt curves plots negative rate of change of fluorescence [-d(RFU)/dT] against temperature.


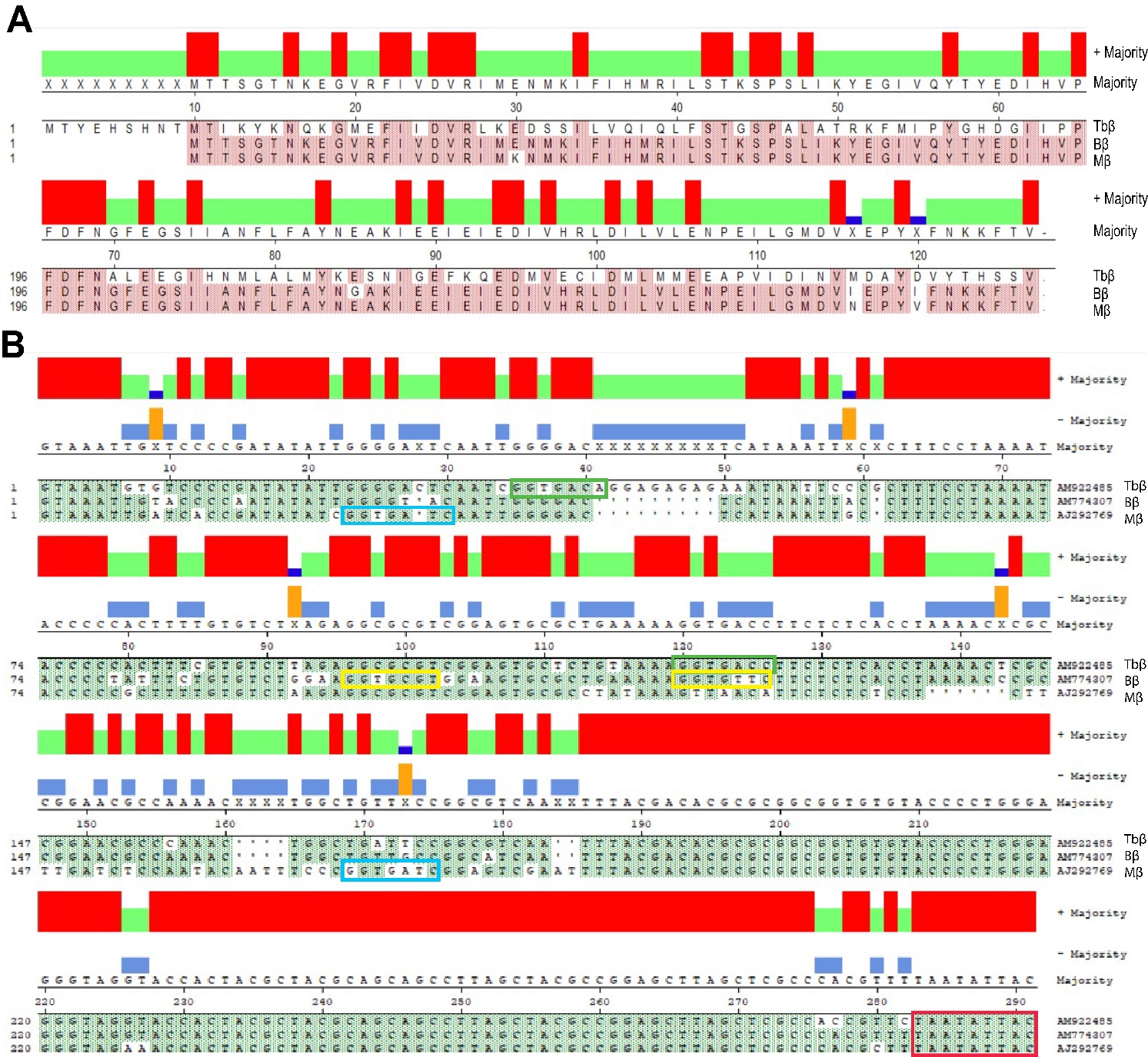


**S3 Fig.** Alignment of coding region βC1 (A) and the satellite conserved region (SCR) (B) from three betasatellites: tobacco leaf curl betasatellite (Tbβ), cotton leaf curl Multan betasatellite (Mβ), and cotton leaf curl Multan betasatellite strain Burewala (Bβ). Sequence similarity is indicated by color: red for highly similar regions across all three betasatellites, green for regions similar in two betasatellites, and blue for dissimilar sequences. Identified repetitive motifs are enclosed in boxes: green for motifs common to all three betasatellites, brown for motifs shared by Tbβ and Bβ, and blue for motifs shared by Bβ and Mβ. Yellow boxes denote previously reported repetitive sequence motifs (Xu et al., 2019). Nonanucleotide sequences are highlighted in red boxes.
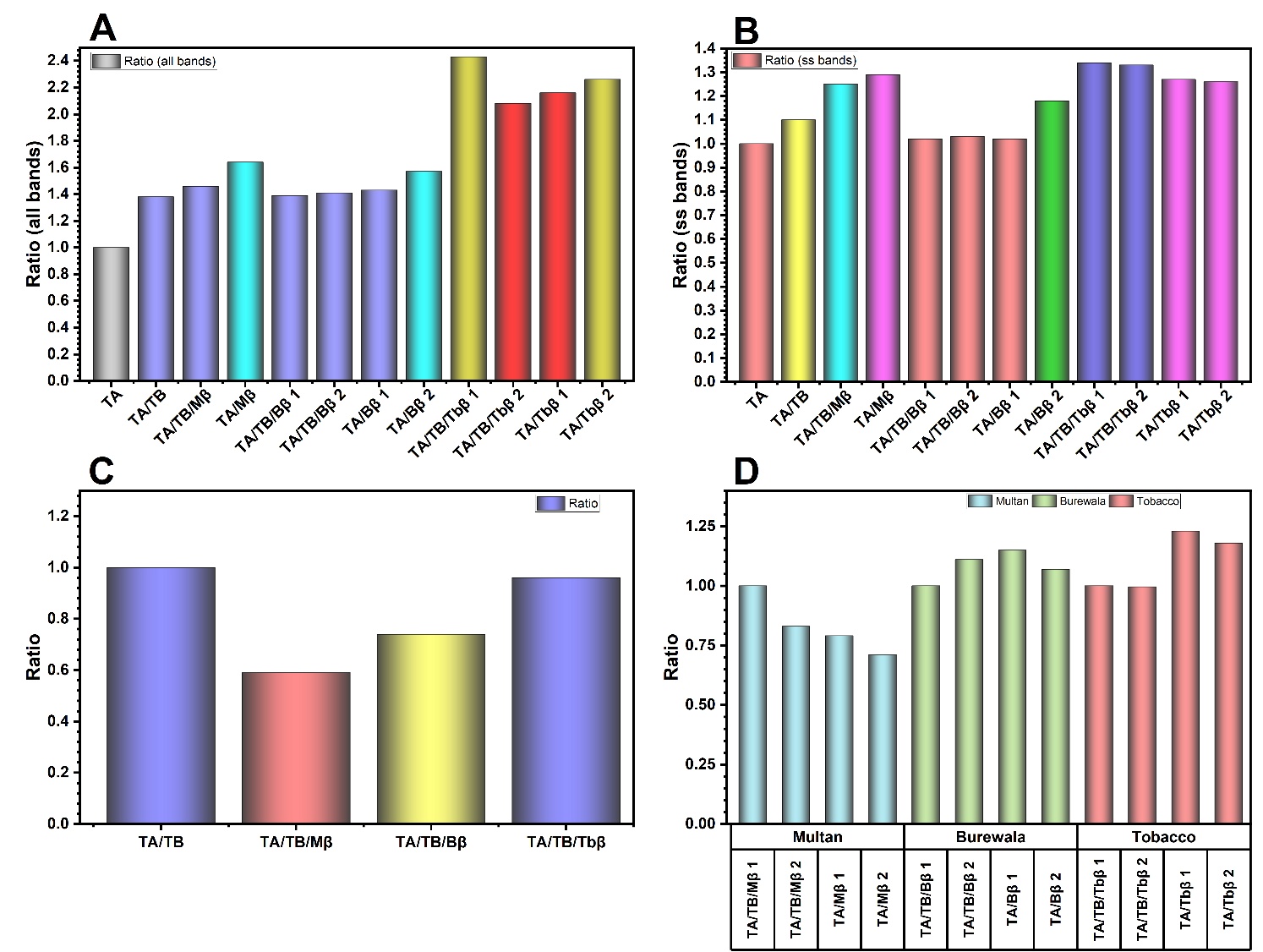


**S4 Fig.** Quantification of Southern blot bands. Relative band intensities (quantification) from Southern blots are shown for ToLCNDV DNA A (A), single-stranded bands of ToLCNDV DNA A (B), ToLCNDV DNA B (C), and three different betasatellites (D). Band intensities were normalized to the corresponding positive control band within each blot, which was set to a value of 1. Panel (D) illustrates the accumulation of three betasatellites: Mβ (yellow), Bβ (blue), and Tbβ (cyan).


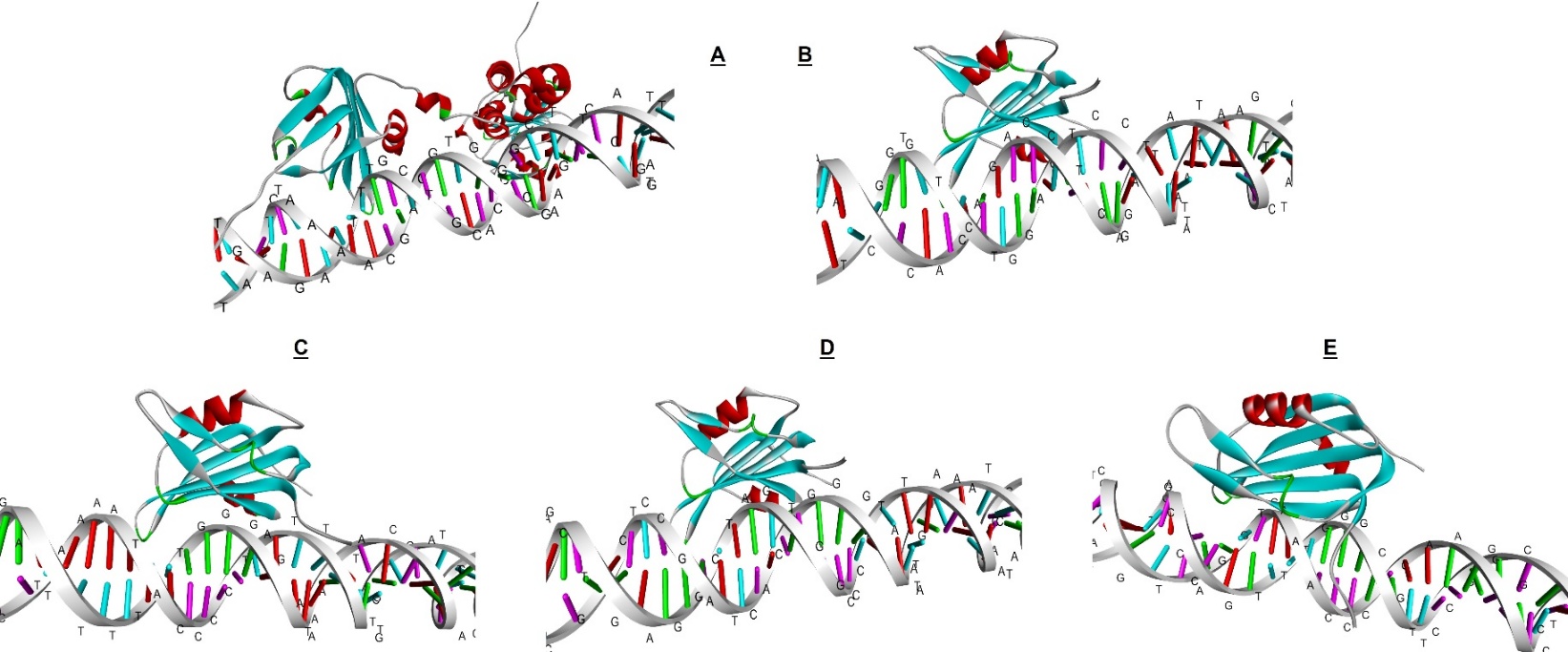


**S5 Fig.** Molecular docking models illustrating the predicted binding interactions between the Rep protein and different DNA molecules: (A) Rep protein docked with CR of DNA-A; (B) Rep protein docked with CR of DNA-B; (C) Rep protein docked with SCR of Bβ; (D) Rep protein docked with SCR of Mβ; (E) Rep protein docked with SCR of Tbβ. Docked complexes are shown as representative structures from the HDOCK server, used for protein-nucleic acid docking analysis.
