# Supplementary material for: Differential interactions of ToLCNDV with different betasatellites reveal complex viral dynamics in N. benthamiana
Source: PLoS One. 2025 Jun 25;20(6):e0327234. doi: 10.1371/journal.pone.0327234 (PMC12193804; doi:10.1371/journal.pone.0327234)

**Raw and uncropped agarose and Southern blot pics**

**Figure 1: ToLCNDV DNA A Gel agarose and Southern blot**

Note (left to right): The gel was loaded with samples in the first 14 wells, followed by two empty wells. A 1ng pGREEN0029 cloning plasmid standard and a 1 kb DNA ladder were then loaded. The two outermost right wells were left empty.


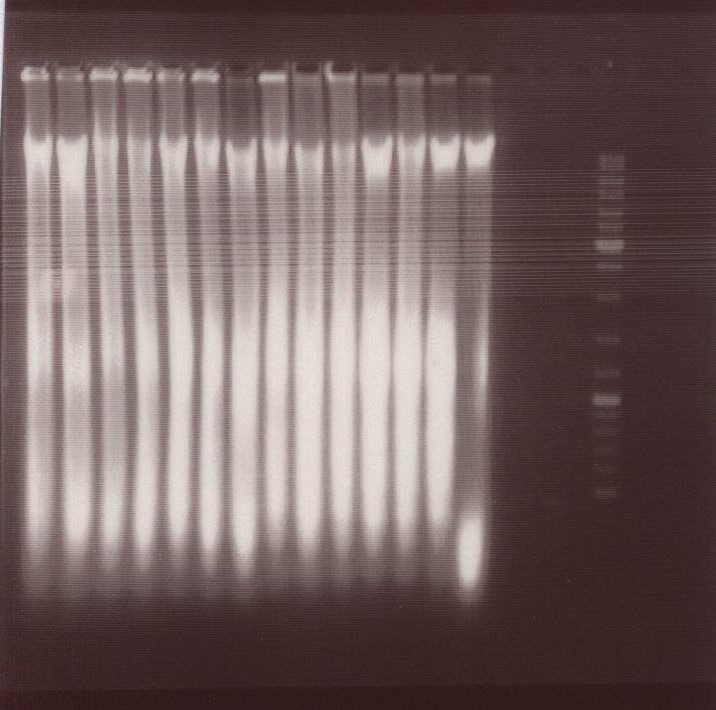


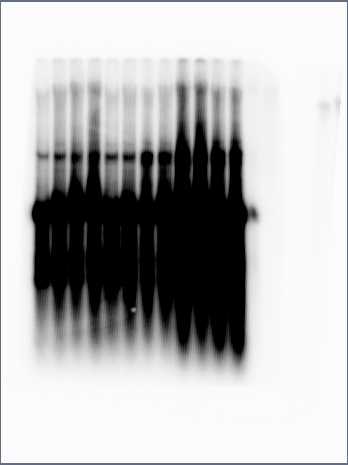


**Figure 2: ToLCNDV DNA B** **Gel agarose and Southern blot**

Note (left to right): The first 10 wells of the gel were loaded with samples, followed by three empty wells. A 1 ng pGREEN0029 cloning plasmid standard was loaded next, then an empty well, and finally a 1 kb DNA ladder.


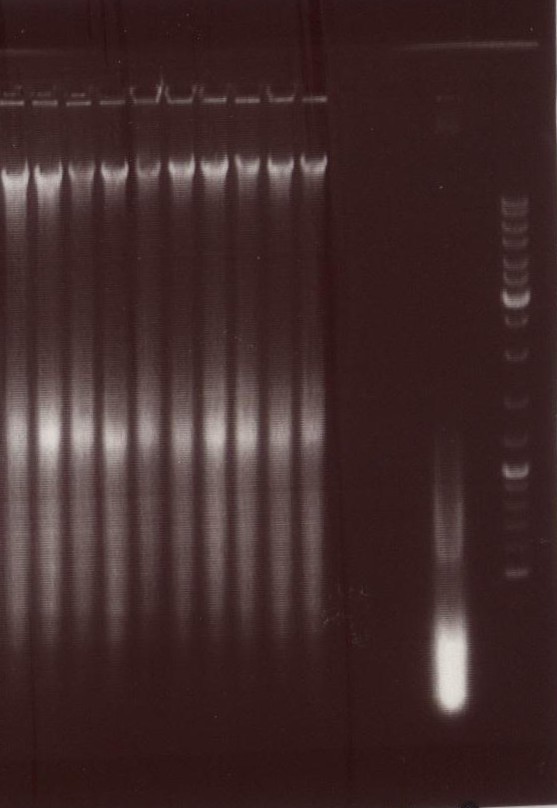


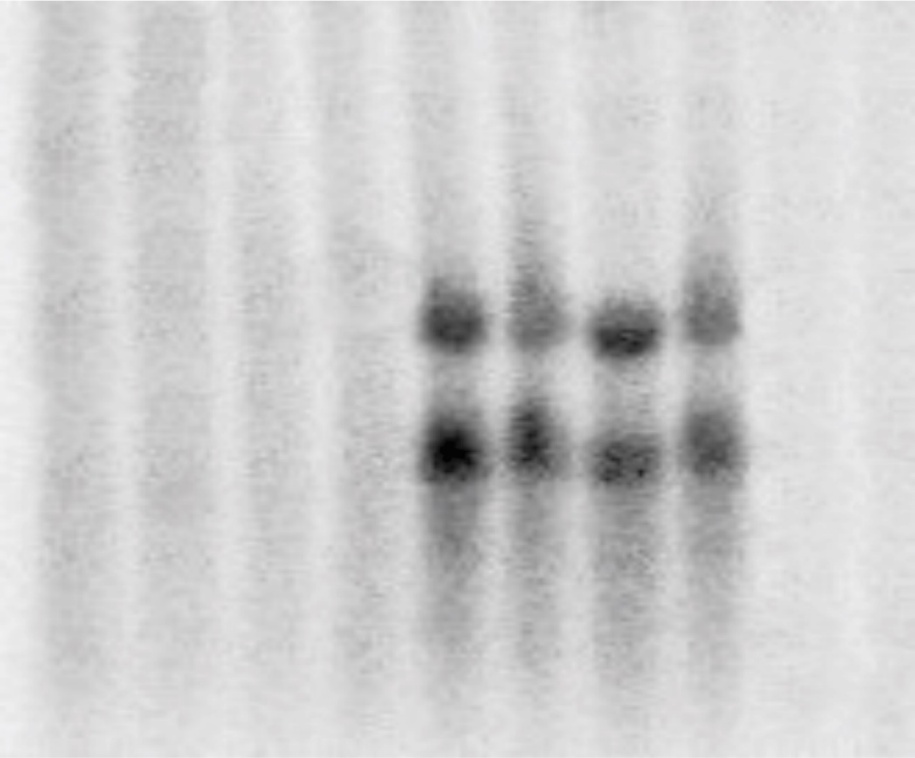


**Figure 3A: CLCuMB (Mβ) agarose gel and Southern blot**

The first 14 wells of the gel were loaded with samples, followed by three empty wells. A 1 kb DNA ladder followed by 1 ng pGREEN0029 cloning plasmid and an empty well.


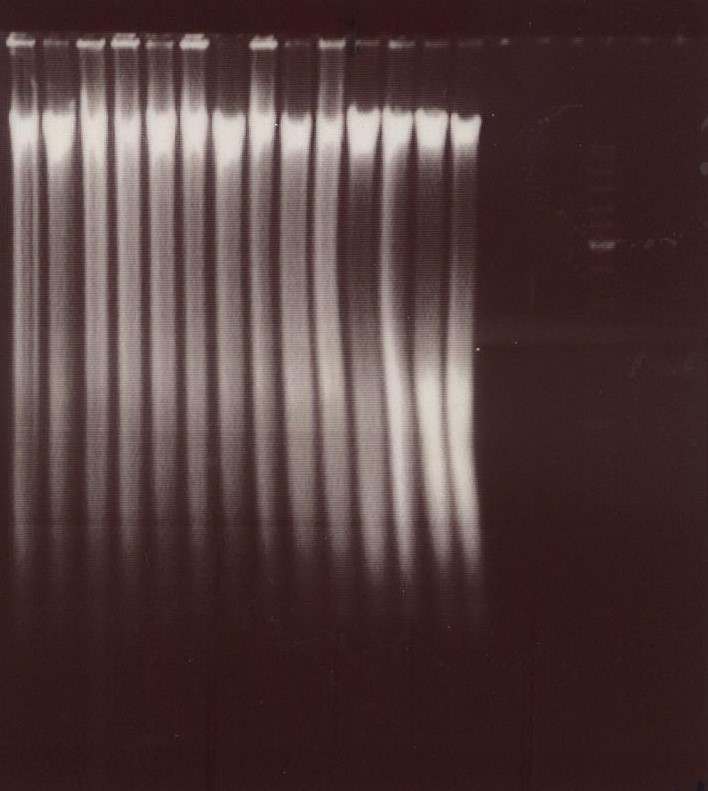


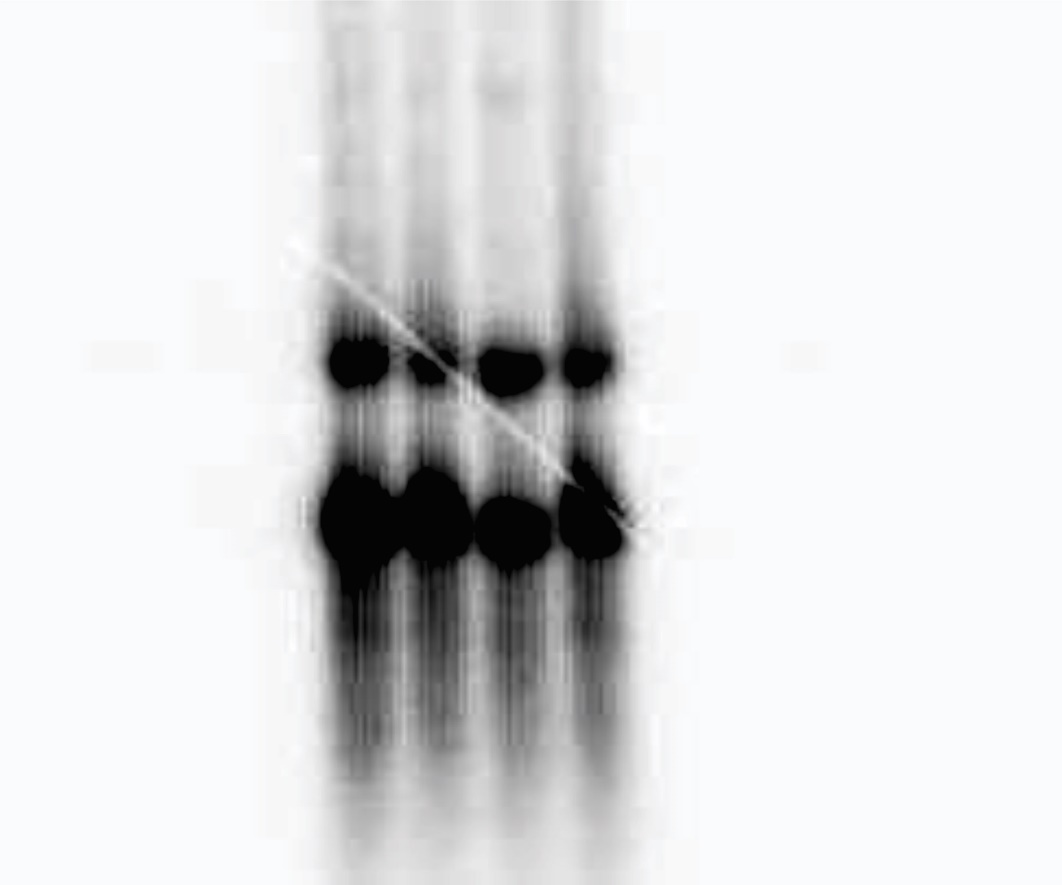


**Figure 3B: CLCuMB^Bur^ (Bβ) agarose gel and Southern blot**

The first 14 wells of the gel were loaded with samples, followed by five empty wells and a 1 kb DNA ladder.


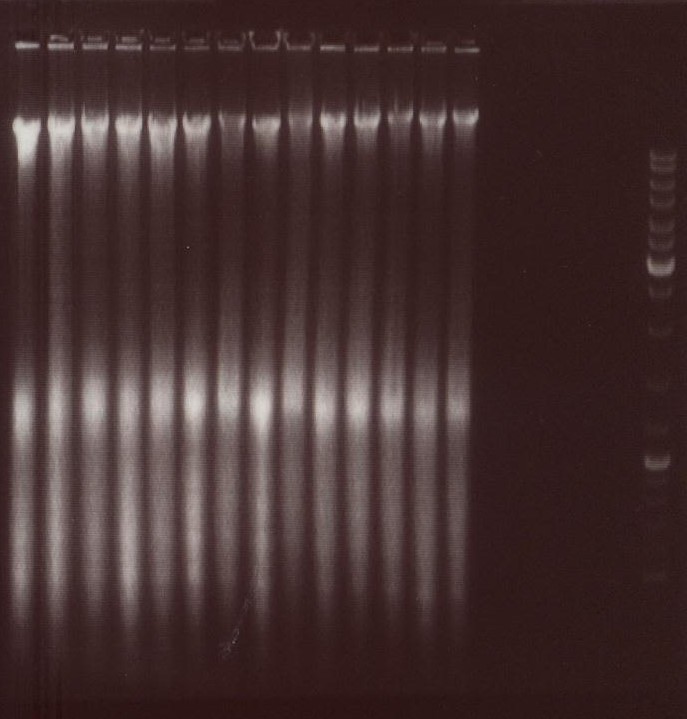


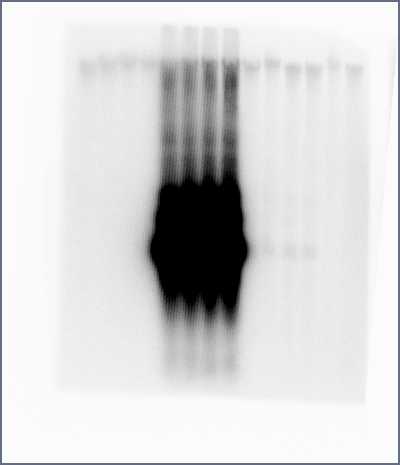


**Figure 3C: TbLCB (Tβ) agarose gel and Southern blot**

The first 14 wells of the gel were loaded with samples, followed by an empty well and a 1 kb DNA ladder.


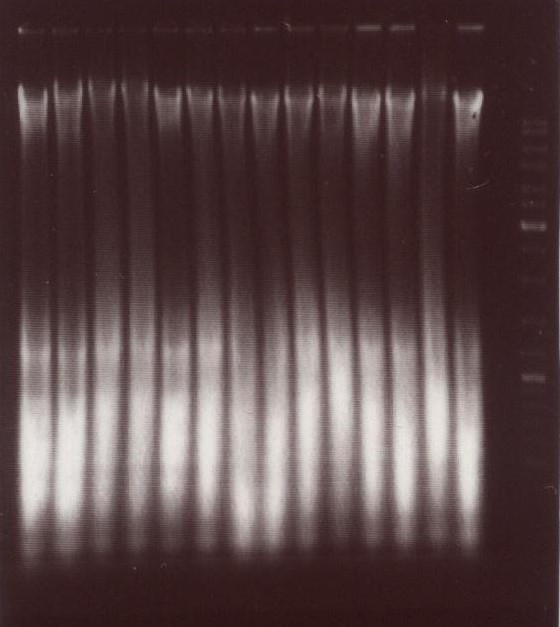


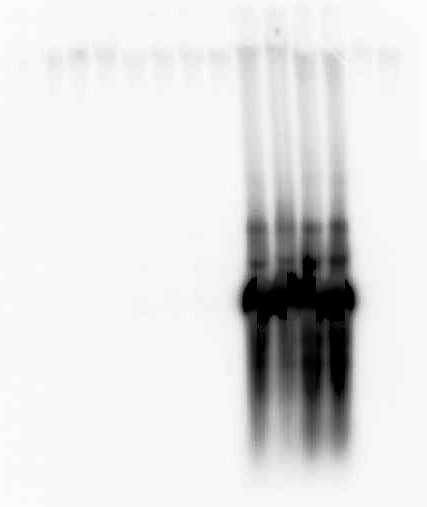

Supplement: S2 File — (DOCX) [file pone.0327234.s002.docx]
