# Supplementary material for: Differential interactions of ToLCNDV with different betasatellites reveal complex viral dynamics in N. benthamiana
Source: PLoS One. 2025 Jun 25;20(6):e0327234. doi: 10.1371/journal.pone.0327234 (PMC12193804; doi:10.1371/journal.pone.0327234)
Supplement: Attachment — Submitted filename: Review 1.28.2025.pdf [file pone.0327234.s003.pdf]

Review of Differential interactions of ToLCNDV with different betasatellites reveal complex viral dynamics in N. benthamiana

This manuscript investigates the interactions between Tomato leaf curl New Delhi virus (ToLCNDV), a bipartite begomovirus, and three betasatellites (cotton leaf curl Multan betasatellite strains Multan [Mβ] and Burewala [Bβ], and tobacco leaf curl betasatellite [Tbβ]) in Nicotiana benthamiana. The study reveals that co-infection with betasatellites alters ToLCNDV dynamics, with Tbβ inducing the highest viral DNA-A (TA) accumulation and more severe symptoms compared to Mβ and Bβ. Southern blotting and qPCR results demonstrate betasatellite-specific influences on viral component replication, suggesting complex and competitive interactions between ToLCNDV DNA-B (TB) and betasatellites for replication machinery. The findings highlight the potential emergence of novel and more pathogenic ToLCNDV-betasatellite combinations in cotton-growing regions, stressing their significant implications for disease management and agricultural productivity. However, there are some questions and concerns that need to be addressed. I suggest major revision.

1. The study proposes competition between DNA-B and betasatellites for TA-encoded Rep. Was the relative binding affinity of Rep to iterons in DNA-B vs. betasatellites experimentally tested (e.g., EMSA, yeast one-hybrid)? This could directly validate the proposed competition.
2. Mβ and Bβ share 96.1% βC1 identity but differ in SCR. How do SCR sequence variations (e.g., non-coding regulatory motifs) influence interactions with ToLCNDV? Were chimeric betasatellites (e.g., swapping SCR regions) tested to isolate SCR-specific effects?
3. Tbβ, originally from soybean, accumulated more efficiently in *N. benthamiana*. Does Tbβ encode solanaceous host-specific adaptations (e.g., promoter elements, βC1 interactions)? Were host-switching experiments conducted (e.g., inoculating soybean with ToLCNDV+Tbβ)?
4. The conclusion questions whether tripartite associations (ToLCNDV + DNA-B + betasatellite) are stable. Were serial passaging experiments performed to track component persistence over time? If not, how confident are the authors in extrapolating field stability?
5. Tbβ caused severe symptoms despite lower TB levels. Is this solely due to higher betasatellite titers, or do βC1 functional differences (e.g., suppression of host defenses) play a role? Were βC1 mutants (e.g., deletion/mutation in silencing suppressor domains) tested?

6. Southern blots detected linear, open-circular, and supercoiled DNA forms. Were these forms quantified separately to assess replication efficiency (e.g., ssDNA as a proxy for active replication)?
7. Symptoms were categorized as "MULC" or "SDLC." Was a standardized scale (e.g., leaf curl index, biomass reduction) used to ensure consistency across replicates/observers?
8. The authors might consider add another protein interaction study. The manuscript suggests competition between TB and betasatellites for replication machinery (e.g., TA-encoded Rep protein). To substantiate this, co-immunoprecipitation (Co-IP) or yeast two-hybrid assays could confirm direct interactions between Rep and betasatellite proteins (e.g., βC1) or DNA.
