# Supplementary material for: Differential interactions of ToLCNDV with different betasatellites reveal complex viral dynamics in N. benthamiana
Source: PLoS One. 2025 Jun 25;20(6):e0327234. doi: 10.1371/journal.pone.0327234 (PMC12193804; doi:10.1371/journal.pone.0327234)
Supplement: Attachment — Submitted filename: Review 03.14.pdf [file pone.0327234.s005.pdf]

Review for Differential interactions of ToLCNDV with different betasatellites reveal complex viral dynamics in N. benthamiana

The authors have addressed many of the concerns raised in my initial review, improving the clarity and depth of their study on *Tomato leaf curl New Delhi virus* (ToLCNDV) interactions with betasatellites. Notably, they have provided additional quantification of Southern blot results, refined their statistical analysis, and enhanced the discussion on ecological relevance. I suggest accepting with major revision.

However, several critical issues remain unresolved, particularly regarding the lack of functional validation for key claims and the absence of serial passaging experiments to confirm tripartite stability. While the study provides valuable insights into ToLCNDV-betasatellite interactions, further revisions are necessary to strengthen the manuscript's conclusions.

1. The authors propose that ToLCNDV's DNA-B and betasatellites compete for the TA-encoded Rep protein, but no direct experimental evidence (e.g., EMSA, yeast one-hybrid, or Co-IP assays) is provided. While they acknowledge this as a limitation, even in silico Rep-binding analysis could help support their hypothesis.
2. The manuscript suggests that ToLCNDV-betasatellite associations may be unstable over time, but this claim is speculative without long-term co-infection studies. The authors reference field reports of ToLCNDV with CLCuMB but do not experimentally test persistence across multiple plant generations. I suggest the authors acknowledge this limitation explicitly and suggest future longitudinal studies.
3. The study focuses solely on ToLCNDV interactions, but a comparison to other bipartite begomoviruses (e.g., *Tomato leaf curl Palampur virus* with betasatellites) would provide a broader perspective. I suggest the authors expand discussion to include known bipartite-betasatellite interactions beyond ToLCNDV.
