# Supplementary material for: Differential interactions of ToLCNDV with different betasatellites reveal complex viral dynamics in N. benthamiana
Source: PLoS One. 2025 Jun 25;20(6):e0327234. doi: 10.1371/journal.pone.0327234 (PMC12193804; doi:10.1371/journal.pone.0327234)
Supplement: Attachment — Submitted filename: Response to Reviewers.docx [file pone.0327234.s006.docx]

***Response to Reviewers***

Authors sincerely thank the reviewers and Editor for their insightful and constructive feedback, which has greatly improved the manuscript. Each comment has been addressed in detail (responses in red font, below), and corresponding revisions are marked with track changes in the manuscript. All line numbers referenced pertain to the track-changed version.

**Editor and Reviewer Comments:**

***EDITOR COMMENTS***

The study was conducted solely on Nicotiana benthamiana, a model plant that may not fully represent the natural hosts of ToLCNDV and its associated betasatellites. This limits the ecological validity of the findings.

Response: The concern regarding the ecological validity of this study is acknowledged, as *Nicotiana benthamiana* is a model plant and may not fully represent interactions in natural hosts. However, its use is justified for initial mechanistic insights and controlled experimentation, providing valuable data on virus-host interactions, viral replication, pathogenicity, and host responses. Additionally, while ToLCNDV is a bipartite begomovirus that typically lacks cognate betasatellites, it uniquely demonstrates the ability to associate with and replicate various betasatellites. This study was designed to assess whether ToLCNDV, recently identified from cotton, could interact with prevalent betasatellites in the region and potentially lead to the emergence of new viral combinations that may threaten cotton productivity.

The study does not appear to include field-based experiments, which are necessary to confirm that these interactions occur under natural conditions, especially in cotton-growing regions where these viruses are prevalent.

Response: While we acknowledge the absence of field-based experiments, crucial for validating virus-betasatellite interactions under natural conditions, this study was designed as a controlled laboratory investigation. The motive was to assess if ToLCNDV along with different betasatellites could lead to the emergence of novel combinations. These controlled conditions minimize environmental variability and provide clear insights into viral replication, pathogenicity, and potential recombination, allowing us to establish the mechanistic basis of ToLCNDV-betasatellite interactions. However, we found that while the apparent antagonism between the betasatellite and DNA-B suggests a nuanced and potentially unstable relationship, it may not necessarily lead to the formation of super virulent tripartite viruses.

Agrobacterium-mediated inoculation does not perfectly mimic natural whitefly-mediated transmission, which could lead to differences in virus accumulation and interactions.

Response: The editor's concern regarding the limitations of *Agrobacterium*-mediated inoculation versus natural whitefly transmission is valid. While *Agrobacterium* infiltration is a widely utilized and efficient method for delivering viral constructs, it may not fully recapitulate the intricate dynamics of whitefly-mediated transmission, which involves specific vector-virus-plant interactions. However, this method provided the necessary information to address our primary objectives, focusing on the mechanistic basis of viral interactions. We acknowledge that future studies incorporating whitefly transmission would enhance the ecological relevance of our findings.

The study relies heavily on qPCR and Southern blot for viral quantification but does not account for potential artifacts like variable DNA extraction efficiency or differential PCR amplification efficiency between different viral components.

Response: This is a valid point, and we took measures to minimize potential artifacts in our qPCR and Southern blot analyses. To mitigate variability in quantification, the same isolated DNA was used for both Southern blot and qPCR. In addition, in qPCR, different biological and technical repeats were used. Furthermore, qPCR conditions, including primer concentrations and thermocycler settings, were rigorously optimized to ensure consistent amplification efficiency across different viral components. We believe these precautions have minimized potential artifacts. This information has been added to the manuscript at line [L291-298] in the revised version.

While the study discusses interactions at a molecular level, there are no functional assays (e.g., protein interaction studies) to confirm the proposed mechanisms.

Response: Authors appreciate the Editor’s point regarding the lack of functional assays. While protein interaction studies would provide deeper mechanistic insights, they are beyond the scope of this manuscript, which primarily focuses on characterizing ToLCNDV and betasatellite interactions *in planta*. However, ToLCNDV-encoded Rep binds to iterated repeat motifs on DNA-B and betasatellites to perform replication. We included this point in the revised script by doing some dry analysis (Figure S4B) based on the previously demonstrated study (Xu et al., 2019).

The study mentions ANOVA and Tukey’s test for data analysis, but it does not provide details on sample size justification or whether the experiments were adequately powered to detect subtle differences.

Response: Authors would like to say that everything asked in the query is clearly mentioned in the script. Please see Section 2.7 and legends of the Figure 5.

The study attributes observed viral titers and symptom severity to interactions between ToLCNDV and different betasatellites but does not explore whether host plant factors (e.g., gene expression differences) contribute to these variations.

Response: We acknowledge the editor's point regarding the role of host factors in begomavirus symptom development. It is well-established that begomaviruses induce symptoms like leaf curling, yellowing, and stunting by disrupting host cellular processes through interactions between viral proteins (Rep, TrAP, C4, etc.) and host factors. Differential expression of these host factors significantly influences symptom severity; for instance, upregulation of defense genes can mitigate symptoms, while downregulation of growth-related genes exacerbates them. Given that we observed consistent phenotypic symptoms across three independent experiments, utilizing the same plant genotype, growth conditions, viral load, and Agrobacterium strain, we anticipate that host factor expression remained relatively stable. Therefore, we attribute the observed symptom differences primarily to the variations in viral components. However, a dedicated study specifically investigating host gene expression profiles in response to the different viral components would be necessary to definitively confirm this. We recognize this as a compelling avenue for future research.

The discussion assumes that ToLCNDV acquiring betasatellites enhances pathogenicity and host adaptation, but it does not investigate whether these interactions provide any evolutionary disadvantages to the virus.

Response: This is a very intriguing point which authors missed thoroughly. Considering the importance of this notion, we discussed this in the light of results in the Discussion section, Please see L442-447 in the revised script.

The study does not appear to include experiments where betasatellites alone were introduced into N. benthamiana without ToLCNDV to determine their independent effects.

Response: It is proven by several studies that betasatellites are true satellites that depend on their helper virus for replication, in plants movement, encapsidation and transmission through insect vectors.

ToLCNDV is studied in isolation with betasatellites, but there is no comparison with other bipartite begomoviruses to determine whether these interactions are unique to ToLCNDV.

Response: The other bipartite begomovirus found infecting different plant species is tomato leaf curl Palampur virus (ToLCPalV), which is found associated with pepper leaf curl betasatellite and CLCuMB in the same region but, notably, this virus is not widespread as ToLCNDV. Additionally, no study has investigated such interactions with ToLCPalV but their natural identification from plant hosts. Nonetheless, authors added this notion to the revised script. Please see L448-456 in the revised script.

The use of Agrobacterium-inoculated infectious clones increases the risk of cross-contamination between viral constructs, potentially leading to misinterpretation of results.

Response: While working with the multiple *Agrobacterium*-inoculated infectious clones could lead to cross-contamination but this wasn’t the case here – as all of our controlled plants remained asymptomatic and didn’t show any PCR-mediated detections.

While primer design is mentioned, the study does not report whether primer specificity was validated against non-target viral sequences to rule out nonspecific amplification.

Response: The used primers were highly specific and yielded only single product – as shown by the melt curve analysis. The same has been mentioned in the revised script (L292-298).

The study does not assess whether ToLCNDV interacting with different betasatellites leads to genetic recombination, which could alter viral evolution and disease dynamics.

Response: This study primarily focused on mechanistic interactions between ToLCNDV and betasatellites, not on assessing recombination. While recombination is a well-documented strategy in begomovirus evolution, it can occur but typically do not occur over just single infection cycles and require sustained co-infections in natural settings. Given the short experimental timeframe, detecting recombination events was beyond this study's scope. However, future longitudinal studies and high-throughput sequencing could help determine whether ToLCNDV-betasatellite interactions drive recombination and impact viral evolution and disease dynamics.

Since ToLCNDV is transmitted by whiteflies, the study should have included experiments evaluating whether these viral interactions influence vector-mediated transmission efficiency.
Response: We acknowledge the editor's point regarding whitefly transmission. While our study focused on *in planta* interactions between ToLCNDV and betasatellites, driven by their co-occurrence and potential for new viral combinations, vector transmission is crucial. Our primary objective was to establish these *in planta* interactions, as they are a prerequisite for efficient transmission. Future studies should incorporate whitefly transmission assays to assess vector acquisition and inoculation efficiencies, providing a more comprehensive understanding of these viral interactions.

***REVIEWER 1***

Major Comments:
1. The study proposes competition between DNA-B and betasatellites for TA-encoded Rep. Was the relative binding affinity of Rep to iterons in DNA-B vs. betasatellites experimentally tested (e.g., EMSA, yeast one-hybrid)? This could directly validate the proposed competition.

Response: Authors appreciate the query as it could provide deeper mechanistic insights into the proposed competition between DNA-B and betasatellites. However, such an investigation is beyond the scope of the current manuscript, which primarily focuses on characterizing the interactions between ToLCNDV and betasatellites *in planta*. Nonetheless, this is a compelling avenue for future research to directly validate the proposed competition and further elucidate the molecular dynamics governing these interactions.

2. Mβ and Bβ share 96.1% βC1 identity but differ in SCR. How do SCR sequence variations (e.g., non-coding regulatory motifs) influence interactions with ToLCNDV? Were chimeric betasatellites (e.g., swapping SCR regions) tested to isolate SCR-specific effects?

Response: The reviewer raises a valid point about the role of the satellite conserved region (SCR) in modulating interactions with Tomato leaf curl New Delhi virus (ToLCNDV), especially given the high βC1 coding region identity (96.1%) between the Mβ and Bβ betasatellites. While chimeric betasatellites, created by swapping SCR regions, would be an ideal approach to directly isolate SCR-specific effects, this study did not initially pursue that avenue.

Instead, following the reviewer's suggestion, we performed a detailed SCR and repetitive motif analysis across the three betasatellites (Tbβ, Mβ, and Bβ) used in this study. This analysis revealed a greater number of shared repetitive sequence motifs between Tbβ and Bβ compared to those shared between Mβ and Bβ. This difference in shared SCR motifs may explain why Bβ exhibited better performance in our assays compared to Mβ, despite the high βC1 sequence similarity. The SCR contains regulatory elements that influence gene expression, replication, and potentially interactions with the helper virus. The observed differences in motif sharing suggest that the SCR of Bβ may contain elements that are more compatible with ToLCNDV or enhance its function in a way that the Mβ SCR does not. Please see the revised version for these updates; MM (Line 126-133), Results (202-211), and Discussion (426-431).

We acknowledge that the chimeric betasatellite approach would provide more definitive evidence of the SCR's specific role. While not performed in this study, the construction and analysis of such chimeric betasatellites represent a compelling direction for future research.

3. Tbβ, originally from soybean, accumulated more efficiently in N. benthamiana. Does Tbβ encode solanaceous host-specific adaptations (e.g., promoter elements, βC1 interactions)? Were host-switching experiments conducted (e.g., inoculating soybean with ToLCNDV+Tbβ)?

Response: Tbβ was originally isolated from the weed *Pedilanthus tithymaloides* but has also been reported to co-infect soybean alongside PedLCV (Ilyas et al., 2010). The reviewer's suggestion regarding Tbβ's host range and potential solanaceous adaptations is indeed compelling and offers a promising avenue for future research. While we appreciate the reviewer's insight, exploring these specific adaptations would necessitate a separate, dedicated study. We believe this constitutes substantial research undertaking in itself and, while highly relevant, falls outside the scope of the present work.

4. The conclusion questions whether tripartite associations (ToLCNDV + DNA-B + betasatellite) are stable. Were serial passaging experiments performed to track component persistence over time? If not, how confident are the authors in extrapolating field stability?

Response: The stability of tripartite begomovirus associations (ToLCNDV DNA-A + DNA-B + betasatellite) under natural conditions, particularly with *Bemisia tabaci* transmission, is a key question, and one we also raised in the Discussion. While serial passaging experiments, which we acknowledge as important, were not performed to directly assess long-term component persistence, existing field evidence supports our assertion of potential stability. The detection of ToLCNDV with CLCuMuB in field-grown cotton (Zaidi et al., 2017) and tomato (Jyothsna et al., 2013) suggests that these tripartite complexes can persist in natural settings. Our finding that ToLCNDV can maintain these three betasatellites (Tbβ, Bβ, and Mβ) further strengthens the likelihood of such stable associations. Furthermore, *B. tabaci*'s polyphagous nature and ability to transmit multiple begomovirus components, along with the natural association of ToLCNDV and CLCuMuB, support this notion. Therefore, while serial passaging experiments would provide more definitive proof, the combined field observations and *B. tabaci* transmission capabilities suggest these associations can persist long enough for detection in diverse hosts.
 We tried to add similar information in the discussion section (Please see L433-438). Hopefully, the kind reviewer will find our explanation satisfactory.

5. Tbβ caused severe symptoms despite lower TB levels. Is this solely due to higher betasatellite titers, or do βC1 functional differences (e.g., suppression of host defenses) play a role? Were βC1 mutants (e.g., deletion/mutation in silencing suppressor domains) tested?

Response: While Tbβ-induced severe symptoms may correlate with its higher betasatellite titers, we cannot rule out potential βC1 functional differences—such as variations in TGS and PTGS—as contributing factors. However, since our study did not focus on βC1 domain mutations or deletions, further investigation would be required to establish a definitive link between βC1 function and symptom severity. However, it is important to mention that a mutational study of βC1 has already been conducted in our lab (DOI: 10.13140/RG.2.2.14851.96804), showing that there was no effect of mutations on βC1 gene when the majority of N- or C‐terminal amino acids were mutated, although one C‐terminal mutant displayed an unusual phenotype. Also one mutation showed a host‐dependant effect, being symptomless in *N. tabacum* and fully symptomatic in *N. benthamiana*.

6. Southern blots detected linear, open-circular, and supercoiled DNA forms. Were these forms quantified separately to assess replication efficiency (e.g., ssDNA as a proxy for active replication)?

Response: The quantification of each band of Southern blot was not detected separately. However, after following the question we quantified the ssDNA of TA DNA and included it in figure S4 and added information in the revised script. Please see L264-267 in the revised version of the script.

7. Symptoms were categorized as "MULC" or "SDLC." Was a standardized scale (e.g., leaf curl index, biomass reduction) used to ensure consistency across replicates/observers?

Response: Actually, no such standard scale is present at global scale and symptoms are demonstrated by phenotypic observation, as we did. However, our lab has devised one in cotton plants and has used it in our previous studies (shafiq et al 2017), as mentioned below.

• Symptom severity scale 0 - No visible symptoms of infection.

• Symptom severity scale 1 - Very mild symptoms consisting of only vein darkening but rarely vein swelling on only a single (the youngest) leaf. Often these symptoms are transient (the plant recovering and showing no further symptoms).

• Symptom severity scale 2 - Symptoms appear late and only the upper leaves are affected. The plants show vein darkening, vein swelling, leaf curling and enations but a little stunting. Rarely plants show the development of leaf-like enations. Plants do not recover.

• Symptom severity scale 3 - Symptoms appear early and most of the plant is affected. The plant shows vein darkening, vein swelling, leaf curling, leaf crumpling and enations as well as stunting. Plants frequently show the development of leaf-like enations. Plants do not recover.

• Symptom severity scale 4 - Plants are extremely stunted with very small, extremely curled leaves with extensive crumpling. Plants appear dark green and do not flower. Leaves show extensive vein darkening, vein swelling and enations, as well as, in many cases, leaf-like enations.

8. The authors might consider add another protein interaction study. The manuscript suggests competition between TB and betasatellites for replication machinery (e.g., TA-encoded Rep protein). To substantiate this, co-immunoprecipitation (Co-IP) or yeast two-hybrid assays could confirm direct interactions between Rep and betasatellite proteins (e.g., βC1) or DNA.

Response: The reviewer's query regarding protein interaction study is well-founded, and we appreciate the suggestion. We did explore repetitive motifs within the sequences (as detailed in our response to Query 2), which potentially include Rep binding sites. However, a dedicated investigation is beyond the scope of this study. While highly relevant and certainly of interest for future work, such an analysis would require a separate, focused investigation.

***REVIEWER 2***

1, the abstract can be shorten.

Response: Authors tried to concise the abstract and reduced it to 228 words.
2, Section 3.3 Quantification of the southern blot is required, as there appears to be a large variation in loading control.

Response: We thank the Reviewer for his valuable suggestion and agree on the importance of quantification. Using the ImageJ program, we have aquantified the Southern blot results (Please see Figure S4). Additionally, as recommended by Reviewer 1, we have quantified the single-stranded DNA bands from the TA Southern blot analysis, and these findings are presented in the updated Figure S4B.

3, Please include all the data points in Figure 6.

Response: We sincerely apologize for the oversight in our initial submission. Upon reviewing the manuscript, we realized there was a misunderstanding regarding the figure numbering. The manuscript contains only five figures, and there is no Figure 6. We have carefully ensured that all relevant data points are included and clearly presented within the existing figures. If there are specific data points or results the Reviewer would like us to highlight or expand upon, we would be happy to provide additional details or clarification. Thank you for bringing this to our attention.
4, Discussion should be more focus on the results but not like a review paper.

Response: We thank the reviewer for this comment. We understand the concern regarding the discussion's focus. Some background context was provided for interpretation, potentially lengthening the discussion, especially related to betasatellites. However, we tried to refine the discussion to sharpen the focus. Please see the revised version of the script.
