# Supplementary material for: Differential interactions of ToLCNDV with different betasatellites reveal complex viral dynamics in N. benthamiana
Source: PLoS One. 2025 Jun 25;20(6):e0327234. doi: 10.1371/journal.pone.0327234 (PMC12193804; doi:10.1371/journal.pone.0327234)
Supplement: Attachment — Submitted filename: Response to Reviewer_R2.docx [file pone.0327234.s007.docx]

***Response to Reviewer_R2***

Authors sincerely thank the reviewer for his insightful and constructive feedback. Authors tried to address each comment in detail (responses in red font, below), and corresponding revisions are marked with track changes in the manuscript. All line numbers referenced pertain to the track-changed version.

**Reviewer Comments:**

***REVIEWER 1***

1. The authors propose that ToLCNDV’s DNA-B and betasatellites compete for the TA-encoded Rep protein, but no direct experimental evidence (e.g., EMSA, yeast one-hybrid, or Co-IP assays) is provided. While they acknowledge this as a limitation, even in silico Rep-binding analysis could help support their hypothesis.

Response: Authors performed Protein-nucleic acid docking studies to verify the hypothesis. All the related sections of the manuscript (MM (L 184-196), Results (L 332-344), and Discussion (L 384-387 and 421-423) have been updated accordingly. All the necessary docking information has been added to the revised Supplementary data.

2. The manuscript suggests that ToLCNDV-betasatellite associations may be unstable over time, but this claim is speculative without long-term co-infection studies. The authors reference field reports of ToLCNDV with CLCuMB but do not experimentally test persistence across multiple plant generations. I suggest the authors acknowledge this limitation explicitly and suggest future longitudinal studies.

Response: Authors acknowledge that this assertion relies on field reports of ToLCNDV co-occurring with CLCuMB without experimental validation of persistence across multiple plant generations. In response, we have explicitly noted this limitation in the revised manuscript (Discussion, L 444-452) and added a recommendation for future longitudinal co-infection studies to assess the stability of these associations over time. These revisions clarify the current evidence gap and outline a path forward to strengthen our findings.

3. The study focuses solely on ToLCNDV interactions, but a comparison to other bipartite begomoviruses (e.g., Tomato leaf curl Palampur virus with betasatellites) would provide a broader perspective. I suggest the authors expand discussion to include known bipartite-betasatellite interactions beyond ToLCNDV.

Response: Authors thank the reviewer for suggesting a broader comparison with other bipartite begomoviruses, such as Tomato leaf curl Palampur virus (ToLCPalV), to contextualize ToLCNDV’s interactions with betasatellites. While our study focuses on ToLCNDV to address its specific prevalence and impact in our sampled regions, we recognize the value of a comparative perspective. In response, we have expanded the Discussion (lines 462–471) to include known bipartite-betasatellite interactions. We appreciate this suggestion and believe it strengthens the manuscript’s contribution to begomovirus research.
